# Complete mitochondrial genome of the Antarctic fur seal (*Arctocephalus gazella*)

**DOI:** 10.1080/23802359.2019.1662751

**Published:** 2019-09-06

**Authors:** Rebecca Nagel, Jaume Forcada, Joseph I. Hoffman

**Affiliations:** a Department of Animal Behaviour, Bielefeld University, Bielefeld, Germany;; b British Antarctic Survey, Cambridge, UK

**Keywords:** Antarctic fur seal, Pinnipedia, Otariidae, *Arctocephalus*, mitogenome

## Abstract

The Antarctic fur seal (*Arctocephalus gazella*) is an abundant Antarctic otariid. Here, we present the complete mitochondrial DNA sequence of this species, which includes 13 protein-coding genes, 22 transfer RNA genes, 2 ribosomal RNA genes, and the control region for a total length of 16,156 bp. A phylogenetic analysis including all 25 publically available pinniped mitogenomes nested the Antarctic fur seal within the Otariid clade, which was clearly resolved from the Phocidae and Odobenidae.

The Antarctic fur seal (*Arctocephalus gazella*) is a marine mammal belonging to the pinniped family Otariidae. It has a circumpolar distribution (Forcada and Staniland 2018), with around 95% of individuals concentrated around South Georgia (Hofmeyr 2016). Despite having experienced a severe demographic decline as a result of industrial-scale hunting in the eighteenth and nineteenth centuries (Hoffman et al. 2011), this species has since staged a remarkable recovery. Today, the breeding population is estimated at several million and is classified as ‘least concern’ by the IUCN (Hofmeyr 2016). Nonetheless, numbers of breeding females at South Georgia have been declining over the past three decades as a result of climate change-driven reductions in food availability (Forcada and Hoffman 2014).

Here, we assembled the complete mitogenome of *A. gazella* using Illumina data (NCBI-SRA: accession number SRR2658532) that were recently used to construct a draft reference genome (Humble et al. 2016). The reads were generated from liver tissue (NCBI-SRA: BioSample SAMN04159679) collected *post mortem* from an adult female that died of natural causes at Bird Island, South Georgia (54°00'24.8ʺS, 38°03'04.1ʺW). Sampling was carried out by the British Antarctic Survey under permits from the Government of South Georgia and the South Sandwich Islands (Wildlife and Protected Areas Ordinance (2011), permit number WPA/201 3/008) and imported to the UK (Department for Environment, Food and Rural Affairs Animal Health Act, import licence number AHZl2024A12005/1; Convention on International Trade in Endangered Species of Wild Fauna and Flora, import number 511446/01). The sample is currently stored in a frozen tissue archive at Bielefeld University. Further information pertaining to DNA extraction, library preparation, Illumina HiSeq 2500 sequencing, and trimming and quality control of the raw reads can be found in Humble et al. (2016).

Quality assessment of the processed reads was performed using FastQC v0.11.8 (Andrews 2010). The full mitogenome was then assembled using MITObim v1.9 (Hahn et al. 2013). We executed three independent assembly runs using the quick function, whereby each run used a different fur seal mitogenome as a bait sequence: *A. pusillus*, *A. townsendi*, or *A. forsteri* (Lin et al. 2002; Arnason et al. 2006). The resulting sequences were aligned using MUSCLE in Geneious Prime v2019.1.3 (https://www.geneious.com) and a consensus sequence was constructed using a 75% base call threshold. MITOS (Bernt et al. 2013) was then used for annotation.

The mitogenome of *A. gazella* is 16,156 bp (GenBank accession number: BK010918) and includes 13 protein-coding genes, 22 transfer RNAs, 2 ribosomal RNAs, and 1 control region, as expected for most vertebrate mitogenomes.

For our phylogenetic analysis, all 25 publically available pinniped mitogenomes and that of the polar bear *Ursus maritimus* (Delisle and Strobeck 2002) were downloaded from EMBL-ENA and aligned using the localpair algorithm in MAFFT v7.4 (Katoh and Standley 2013). We ran jModelTest v2.1.10 (Darriba et al. 2012) for model selection; BIC and AIC found the GTR + I+G substitution model to be the best model. This model was used to construct a phylogenetic tree using RAxML v8.2.12 (Stamatakis 2014) with 500 bootstrap replicates (Figure 1).

**Figure 1.**
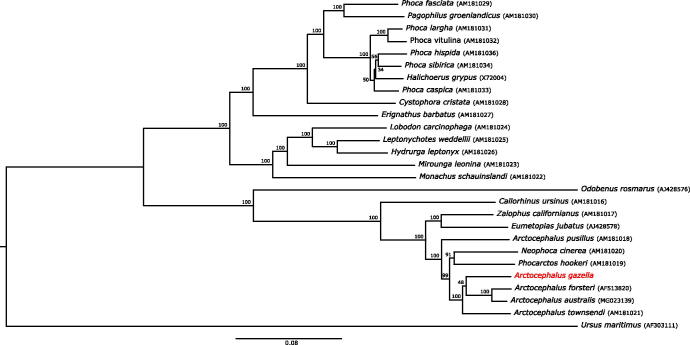
A maximum-likelihood phylogenetic tree based on the complete mitochondrial genomes of 26 pinniped species, including representatives from all three extant families (the Phocidae, Odobenidae and Otariidae). The polar bear *Ursus maritimus* was used as the outgroup. Species names are given together with GenBank accession numbers in parentheses. Bootstrap values are shown for each node.
